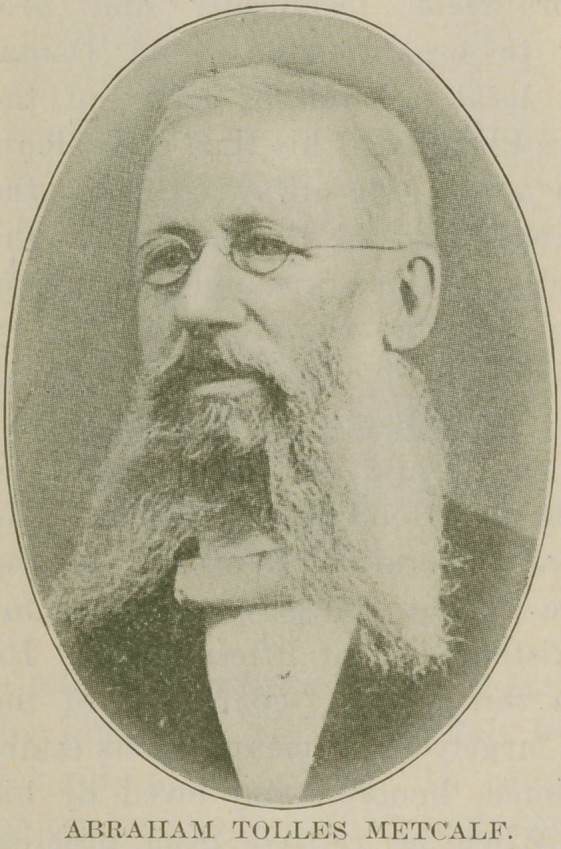# Abraham Tolles Metcalf, D.D.S.

**Published:** 1916-11

**Authors:** 


Abraham Tolles Metcalf, D.D.S. Dr. Metcalf was born February 26, 1831, in Whitestown, N. Y. He died October 28, 1916, in Battle Creek, Mich. He began to practice dentistry in Utica, N. Y., in 1851. In 1853 he moved to Kalamazoo, Michigan, where he practiced continuously until he retired from practice and moved to Battle Creek in 1890.

Dr. Metcalf was a pioneer practitioner in many respects. He entered the profession when the working equipment of a dentist was very meager. He made all his own operating instruments. His pluggers for using crystal gold he made. In order to enlarge the handles of these instruments so that he could use more power by hand in condensing his crystal gold he placed thick leather washers over the handle of the instruments to enlarge them especially near the distal ends so that by placing the leather-made end in the palm of his hand he could apply a maximum of force in condensing the gold. When the cohesive property of gold was discovered by annealing it, Dr. Metcalf made the first alcoholic lamp for annealing gold. He also invented the first dental engine which was run by pneumatic power. The mechanic who helped him in the construction of his pneumatic engine afterwards discovered and made the first electric mallet with the help of Dr. Metcalf.

ABRAHAM TOLLES METCALF.

Dr. Metcalf assisted in organizing the first dental society in the state of Michigan in 1855. He was always actively engaged in society work during the time he was in practice, and in fact never lost his interest in the profession. In the year 1874 and '75 he was a member of the Michigan Legislature, and while there he got a law passed appropriating money for the establishment of a dental department in connection with the University of Michigan. He has maintained a continued interest in the dental department all the years since it was organized. He wrote the first paper ever written on the "Use of Crystal Gold," it was published in 1851 in the *Jones, White and McCurdy Dental Journal.* Dr. Metcalf was a man of character and strong convictions which occasionally made his life somewhat strenuous. He served on the Michigan State Board of Dental Examiners for two or three terms, during the formation period of dental legislation, and always with a proper appreciation of the important responsibilities involved.

He was not an extensive contributor to dental literature but he wrote several papers of value and always supported the literary program of the dental societies in discussions of papers read. He did not identify himself intimately with the work of the National Dental Society, but was a frequent attendant. He did much to encourage higher standards of education through laws regulating the practice of dentistry in Michigan, even after he had permanently retired from practice.

He was one of the oldest and most active members of the Masonic Fraternity of Michigan; in fact he was one of the few masons to have received the highest degree conferred by the Masonic Fraternity on any one.

He has had several political honors conferred by his fellow citizens. He was respected for his strong character and high moral life. His life of devotion to his profession made him worthy of our admiration and esteem. He did much for his profession and humanity is the gainer because he lived and served faithfully and well.

## Figures and Tables

**Figure f1:**